# Personal characteristics and transmission dynamics associated with SARS-CoV-2 semi-quantitative PCR test results: an observational study from Belgium, 2021–2022

**DOI:** 10.3389/fpubh.2024.1429021

**Published:** 2024-09-10

**Authors:** Toon Braeye, Kristiaan Proesmans, Dieter Van Cauteren, Ruben Brondeel, Niel Hens, Elias Vermeiren, Naïma Hammami, Angel Rosas, Adrae Taame, Emmanuel André, Lize Cuypers

**Affiliations:** ^1^Epidemiology of Infectious Diseases, Sciensano, Brussels, Belgium; ^2^I-BioStat, Data Science Institute, Hasselt University, Hasselt, Belgium; ^3^Faculty of Pharmaceutical Sciences, Department of Bio-analysis, Ghent University, Ghent, Belgium; ^4^Centre for Health Economic Research and Modelling Infectious Diseases, Vaccine and Infectious Disease Institute, University of Antwerp, Antwerp, Belgium; ^5^Department of Care, Infection Prevention and Control, Flemish Community, Brussels, Belgium; ^6^Direction Surveillance des Maladies Infectieuses, Agence out une Vie de Qualité (AVIQ), Charleroi, Belgium; ^7^Cellule de médecine préventive- Direction santé et aide aux personnes – Vivalis/Cocom, Brussels, Belgium; ^8^Department of Laboratory Medicine, National Reference Centre for Respiratory Pathogens, University Hospitals Leuven, Leuven, Belgium; ^9^Department of Microbiology, Immunology and Transplantation, Laboratory of Clinical Microbiology, KU Leuven, Leuven, Belgium

**Keywords:** SARS-CoV-2, epidemiology, viral load, transmission, vaccine effectiveness, contact tracing

## Abstract

**Introduction:**

Following harmonization efforts by the Belgian National Reference Center for SARS-CoV-2, semi-quantitative PCR test (SQ-PCR) results, used as a proxy for viral load, were routinely collected after performing RT-qPCR tests.

**Methods:**

We investigated both the personal characteristics associated with SQ-PCR results and the transmission dynamics involving these results. We used person-level laboratory test data and contact tracing data collected in Belgium from March 2021 to February 2022. Personal characteristics (age, sex, vaccination, and laboratory-confirmed prior infection) and disease stage by date of symptom onset were analyzed in relation to SQ-PCR results using logistic regression. Vaccine effectiveness (VE) against a high viral load (≥10^7^ copies/mL) was estimated from the adjusted probabilities. Contact tracing involves the mandatory testing of high-risk exposure contacts (HREC) after contact with an index case. Odds ratios for test positivity and high viral load in HREC were calculated based on the SQ-PCR result of the index case using logistic regression models adjusted for age, sex, immunity status (vaccination, laboratory-confirmed prior infection), variant (Alpha, Delta, Omicron), calendar time, and contact tracing covariates.

**Results:**

We included 909,157 SQ-PCR results of COVID-19 cases, 379,640 PCR results from index cases, and 72,052 SQ-PCR results of HREC. High viral load was observed more frequently among recent cases, symptomatic cases, cases over 25 years of age, and those not recently vaccinated (>90 days). The vaccine effectiveness (VE) of the primary schedule in the first 30 days after vaccination was estimated at 47.3% (95%CI 40.8–53.2) during the Delta variant period. A high viral load in index cases was associated with an increased test positivity in HREC (OR 2.7, 95%CI 2.62–2.79) and, among those testing positive, an increased likelihood of a high viral load (OR 2.84, 95%CI 2.53–3.19).

## 1 Introduction

The first surge of SARS-CoV-2 infections, which led to coronavirus disease 2019 (COVID-19), severely strained Belgium’s healthcare system from March to May 2020 and resulted in considerable excess mortality (1–3). In response, laboratory case surveillance was expanded, and contact tracing efforts were centralized to control the spread of SARS-CoV-2 (4). Centralized contact tracing in Belgium, which involved forward-tracing all high-risk exposure contacts (HREC) of confirmed, cases was implemented from May 2020 to April 2022. We previously described the contact tracing process and its main performance metrics (5, 6).

In short, contact tracing was initiated when a person without a recent history of SARS-CoV-2 infection tested positive. These individuals, referred to as index cases, were interviewed by call centers and asked to report recent contacts. If an exposure was considered high-risk (e.g., direct physical contact, face-to-face contact at a distance of < 1.5 m without a face mask for over 15 min) (7), the HREC were required to undergo PCR testing and remain in quarantine. With the exception of periods when high incidence exceeded the capacity of call centers, such as in October 2020 and January 2022, the system consistently performed well, successfully contacting 72% of index cases and testing approximately 70% of identified HREC in 2021. After 10 January 2022, testing was limited to symptomatic HREC. Contact tracing within collectivities such as hospitals, schools, and long-term care facilities was organized through separate systems (8).

As a laboratory-confirmed infection was a necessary part of the case definition, contact tracing and case surveillance were sensitive to laboratory testing methods. The primary laboratory testing method for detecting SARS-CoV-2 was an RT-PCR test on a nasopharyngeal swab. This approach was inferred to have very high clinical sensitivity to detect viral RNA in individuals (9). In Belgium, centralized databases initially only collected the test result: negative, positive, or indeterminate, which reflected only the presence or absence of viral RNA, without differentiating the amount of RNA detected or distinguishing between a replication-competent virus or residual RNA. A positive test, therefore, does not necessarily reflect a recent infection or infectiousness (10). Research has suggested complementing binary test results with proxy metrics for viral load to stratify transmission risk and infectivity (11, 12). Viral load, as determined by RT-PCR, is either expressed as the number of viral RNA copies per milliliter of viral transport medium or per swab or by the arbitrary test-specific cycle threshold (Ct) value (13). As the number of copies/mL declines exponentially, the PCR Ct-values increase linearly (10). Raw Ct-values are typically hard to interpret from a centralized database as they are sensitive to the storage, preparation, and tests used by the different laboratories.

Belgium’s national reference center (NRC) for SARS-CoV-2 harmonized RT-PCR results by introducing four semi-quantitative categories (SQ-PCR) based on RNA copies/mL. To implement this, the NRC shipped pre-quantified control material to 124 laboratories with instructions to set up a standard curve to define thresholds per assay. This effectively linked a laboratory’s Ct-values to the cut-off concentration of RNA copies/mL. The following categories were defined (RNA copies/mL): weakly positive (<10^3^), moderately positive (10^3^–10^5^), highly positive (10^5^–10^7^), and very highly positive (≥10^7^). The cut-offs and their proposed interpretation were based mostly on *in-vitro* studies, as little epidemiological research was available at the time of design, October 2020. These categories were to be routinely reported as part of the laboratory results. Data on raw Ct values was not routinely collected. While SQ-PCR results should be interpreted in combination with clinical information (14), a weakly positive SQ-PCR can be found at both the start and end of an infection period (15, 16), SQ-PCR results could be compared over different laboratories after this harmonization.

The NRC defined objectives for the harmonized PCR results: to improve reporting, to help clinicians identify the disease stage, to follow-up long-term SARS-CoV-2 positive cases, and to help identify cases with a high transmission risk and samples suitable for whole genome sequencing. The epidemiological significance of the obtained SQ-PCR results, however, has not been systematically explored.

## 2 Materials and methods

### 2.1 Data sources and study period

In this retrospective cohort study, we combined data on SQ-PCR test results with vaccination and contact tracing data. Data on laboratory tests (type of test, test result, sampling date, SQ-PCR) were obtained from the exhaustive Belgian laboratory test database, data on vaccination (date of vaccination, number of doses) from the Belgian COVID-19 vaccination registry Vaccinnet+, data on contact tracing (date of contact, household-membership and linking index cases to HREC, symptoms) from the Belgian contact tracing database (5) and data on personal characteristics (age, sex) from the national registry. Data was stored by Healthdata.be at Sciensano, the National Institute of Health (2). All data was person-level data that could be linked using a unique pseudonymized identifier. Records with missing variables were excluded.

The study period started when the harmonized SQ-PCR results became available on 29 March 2021 and ended on 22 February 2022. On that date, the testing and tracing strategy of HREC underwent a major reform (recommendation to use self-tests after high-risk contact, no more mandatory quarantine). Three different variants of concern (VOC) were dominant, detected in 70% of the samples sequenced by the baseline genomic surveillance (17) during the study period. For the Alpha-VOC, the dominance period started on 29 March 2021 (the start of the study period) and ended on 18 May 2021. Dominance of the Delta-VOC started on 6 July 2021 and ended on 27 December 2021. Omicron dominance started on 4 January 2022, and the study period ended on 22 February 2022. The periods in between VOC dominance were referred to as transition periods (18).

Vaccines used during the study period were ChadOx1 (AstraZeneca®) vaccine and COVID-19 Ad26.CoV2.S (Janssen®) vaccine for primary vaccination and mRNA-1273 (Moderna®) and BNT162b2 (Pfizer®) for primary and booster vaccination. Observations from vaccine administration to when the vaccine was considered effective (7 days for an mRNA vaccine, 14 days for ChadOx1, and 21 days for Ad26.CoV2.S) were not included in the analysis.

### 2.2 Additional covariates

Only SQ-PCR results were available for analysis; corresponding Ct values were not collected. For inclusion of SQ-PCR results as an outcome in logistic regression and in graphs, we have dichotomized the SQ-PCR result into HVL (high viral load, ≥ 10^7^ RNA copies/mL) and not-HVL (< 10^7^ RNA copies/mL), which was in accordance with the highest cut-off suggested by the national reference center (very highly positive).

To explore the person-level temporal dynamics, the proportion HVL was plotted over the number of days since symptom onset. In addition, the period around the symptom onset was categorized into disease stages: presymptomatic (before symptom onset), symptomatic (first 10 days after symptom onset), and late symptomatic (over 10 days after symptom onset). The category ‘asymptomatic’ was assigned to individuals who reported no symptoms after inquiry. When no information was available, the assigned disease stage was ‘unknown.’ The cut-off of 10 days after symptoms (symptomatic period) was set after studies reported that infectious viruses can be isolated for up to 8 or 10 days (10). In Supplementary Table S1, we included a within-person analysis of HVL and disease stages.

### 2.3 Analyses

#### 2.3.1 SQ-PCR and personal characteristics

The logistic regression model for estimating the probability of HVL included the following variables: age group (calculated from the age at the time of testing), sex (as recorded in the national registry), dominant VOC, and calendar week. A person’s immunity status was included as the number of vaccine doses, time since the last dose (effective-30, 30–90, and > 90 days), prior (>60 days) laboratory-confirmed infection, and time since most recent infection (61–120, >120 days).


$$\begin{array}{l} P\left(HVL\right)\sim \mathrm{age}+\mathrm{sex}+VOC+\mathrm{immunity}.\mathrm{status}*VOC\\ \;+\mathrm{disease}.\mathrm{stage}+\mathrm{calendar}.\mathrm{week} \end{array}$$

From this model, vaccine effectiveness (VE) against high viral load given a positive test was estimated. For VE, we limited the analysis to persons without a laboratory-confirmed prior infection.


$$\begin{array}{l} VE_{HVL}=\\ 1-\frac{P\left(HVL|\mathrm{vaccinated}\&\mathrm{no}\;\mathrm{lab}.\;\mathrm{confirmed}\;\mathrm{prior}\;\mathrm{infection}\right)\;}{P\left(HVL|\mathrm{unvaccinated}\&\mathrm{no}\;\mathrm{lab}.\;\mathrm{confirmed}\;\mathrm{prior}\;\mathrm{infection}\right)} \end{array}$$

Parametric bootstrap methods were used to obtain 95% confidence intervals for $VE_{HVL}$ . The proportion of cases with HVL is presented together with the Rt as estimated from the case incidence. To estimate Rt, we used the methods developed by Cori et al. (19) using an average serial interval of 4.7 days and a standard deviation of 2.9 days.

#### 2.3.2 SQ-PCR and transmission

An HREC was considered positive if there was at least one positive laboratory test (PCR or antigen test) in a period of up to 20 days after contact with the call center. HREC without test results were excluded.


$$\begin{array}{l} P\left(\mathrm{positive}\;\mathrm{HREC}\right)\sim SQ-PCR_{\mathrm{index}}+\mathrm{ag}e_{\mathrm{index}}+sex_{\mathrm{index}}\\ +\mathrm{immunity}.\mathrm{statu}s_{\mathrm{index}}*VOC+\mathrm{disease}.\mathrm{stag}e_{\mathrm{index}}+\mathrm{ag}e_{\mathrm{hrec}}\\ +sex_{\mathrm{hrec}}+\mathrm{immunity}.\mathrm{statu}s_{\mathrm{hrec}}*VOC+VOC+\mathrm{calendar}.\mathrm{week}\\ +\mathrm{household}+N.\mathrm{Exposures}+N.\mathrm{HREC}+\mathrm{CIAH} \end{array}$$

We adjusted for personal characteristics (age and sex), dominant VOC, calendar week, and immunity status. To maintain model stability, the immunity status was included without the time since the most recent prior laboratory-confirmed infection. Finally, different contact-tracing-specific variables were included. Previous model-building efforts have indicated the importance of these variables, and we consider them to be potential confounders of this analysis. Household was a binary variable to indicate if the HREC and index case were part of the same household. CIAH defined if the index case had previously been identified as HREC (in the 20 days prior to being contacted as an index case). N.Exposures defined the number of times an HREC has been contacted by the call center in the previous 20 days for different index cases. N.HREC defined the number of HREC reported by the index.

To investigate the transmissibility of SQ-PCR results between index cases and HREC, we fitted the logistic regression model to positive HREC with an SQ-PCR result: $P\left(HVL\;\mathrm{HREC}\right)\sim SQ-PCR_{\mathrm{index}}$ . The covariates included were equal to the previous analysis. If multiple SQ-PCR results were available for the transmissibility analysis, of which one was HVL, we included the HVL result. The impact of this decision is limited. This is further explored in the Supplementary materials, in which we also included additional sensitivity analyses. We refitted the transmission model with data limited to contacts within or outside of the household.

#### 2.3.3 Model fitting and software

All analyses were performed in R software version 4.2.1 (20).

## 3 Results

### 3.1 Numbers included

Over the study period, 2,875,675 positive (PCR and antigen) tests were reported for 2,633,974 cases, of which 92% were PCR tests. For 916,126 tests (32%), SQ-PCR results were available. After the removal of records with missing variables (age, sex, sampling date, immunity status), 909,157 records could be included in the regression (99% of all records with SQ-PCR results). Symptomatic presentation was unknown for 25% of the cases, while 27% of the cases included reported no symptoms.

For the transmission analysis, 390,563 HREC (26% of all tested HREC during the study period) had an index case with an available SQ-PCR result. For 75,272 contacts, both HREC and index cases had SQ-PCR results. After the removal of records with missing variables, we included 379,640 HREC (97% of all tested HREC with index case with SQ-PCR result), of which 139,056 (37%) tested positive and 72,052 (19%) had SQ-PCR results.

The majority of cases reported during the study period had no SQ-PCR results. We explored the potential impact of this in the Supplementary materials and found no evidence of selection bias. Laboratories either reported SQ-PCR results for all cases or for none. We found no evidence of selective reporting by laboratory. A description of the study population (age, sex, vaccination, and prior infection) is presented as Supplementary Figure S5.

### 3.2 SQ-PCR and personal characteristics

**Figure 1:**
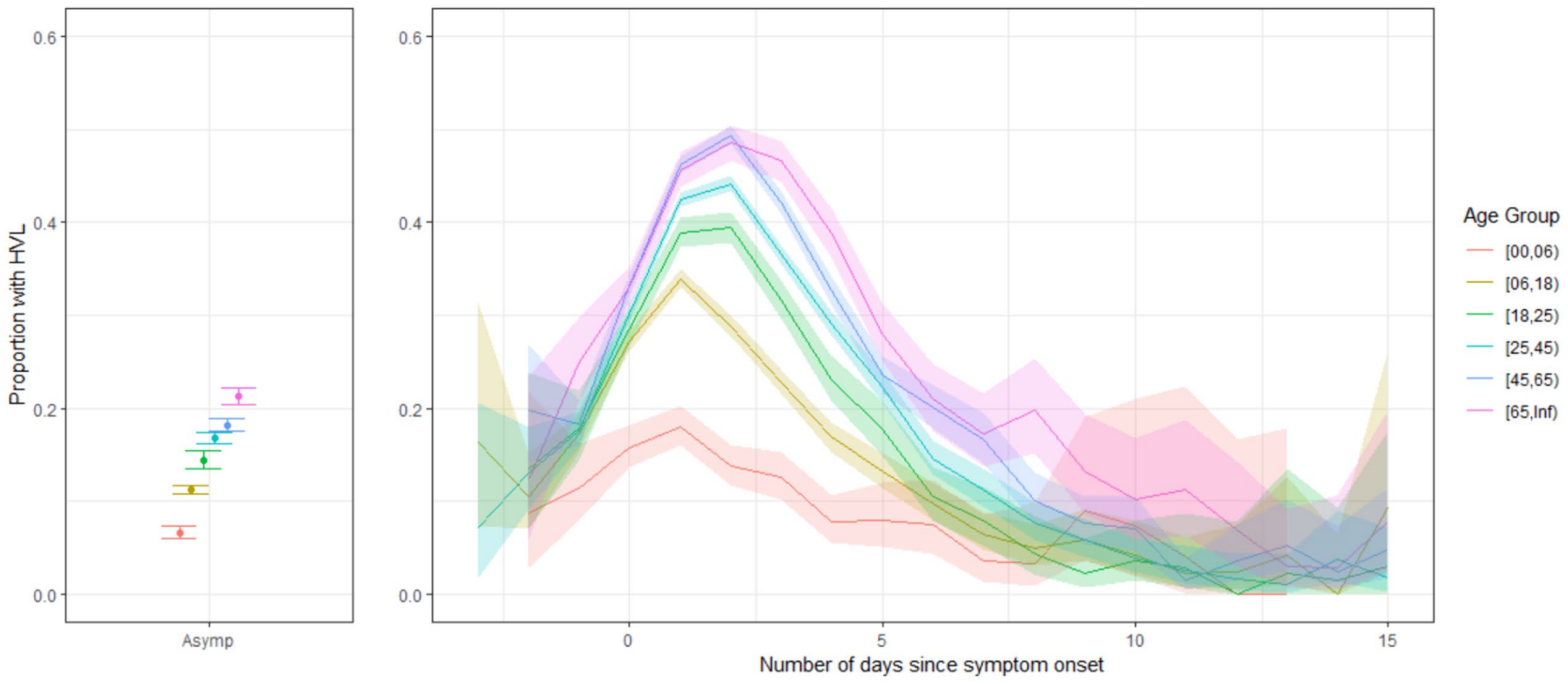
The proportion of cases with high viral load (HVL) over all cases with SQ-PCR results for asymptomatic cases and symptomatic cases by the number of days since symptom onset by age group, during the period of Delta dominance, Belgium, 6 July 2021–2027 December 2021.

HVL was observed more often in symptomatic cases: 28% in symptomatic cases versus 15% in asymptomatic cases (Table 1). Overall, the proportion of HVL was highest shortly after symptom onset (Figure 1). Through multivariate logistic regression, we obtained a significant association between HVL and the first 10 days after disease onset (OR 2.29 (95% CI 2.25–2.32), reference: asymptomatic) (Table 2). A late symptomatic period (>10 days after symptom onset) was typically associated with significantly lower odds of HVL (OR 0.64 (95% CI 0.61–0.69), reference: asymptomatic). For the presymptomatic period, the results of the fixed effects model [OR 1 (95% CI 0.94–1.05)] and the results of the within-person analysis (mixed effects model OR 1.44 (95% CI 1.14–1.81), Supplementary Table S1) pointed to equal or higher odds compared to asymptomatic. The OR for HVL significantly increased with age. In persons under the age of 25, a much lower proportion of HVL was observed compared to persons aged 25 to 44 years (Table 2; Supplementary Figure S1).

**Table 1:** Case numbers by disease stage and SQ-PCR result.

	Weakly positive (14%)	Moderately positive (20%)	Highly positive (44%)	Very highly positive (HVL) (22%)	Unknown
Asymptomatic	46,104 (27%)	42,012 (25%)	56,042 (33%)	24,948 (15%)	408,810
Presymptomatic	2,494 (24%)	2,397 (23%)	3,925 (37%)	1,717 (16%)	19,292
Symptomatic	29,658 (6%)	60,566 (13%)	245,356 (52%)	133,396 (28%)	780,974
Late symptomatic	9,128 (49%)	4,539 (24%)	3,754 (20%)	1,280 (7%)	4,3,491
Unknown	42,309 (17%)	75,429 (30%)	91,013 (37%)	40,059 (16%)	700,983

Cases have both a self-reported disease stage [presymptomatic (1%), symptomatic (44%), late symptomatic (2%), asymptomatic (20%), and unknown (33% of all included cases)] and a corresponding SQ-PCR result. Percentages in the table present proportions across rows among those with a reported SQ-PCR result (HVL, high viral load; SQ, semi-quantitative), Belgium, 29 March 2021–22 February 2022.

**Table 2:** Odds ratios and lower and upper bounds of the 95% CI from the analysis on personal and temporal characteristics for high viral load (reference: positive test without high viral load).

Variable	Level	Odds ratio	Lower bound	Upper bound
VOC	Alpha	ref		
	Delta	0.99	0.83	1.17
	Omicron	1.13	0.96	1.33
	transit	1.17*	1.00	1.36
Age group	[00,06)	0.33*	0.32	0.35
	[06,18)	0.74*	0.73	0.75
	[18,25)	0.93*	0.92	0.95
	[25,45)	ref		
	[45,65)	1.12*	1.11	1.14
	[65, Inf)	1.31*	1.28	1.33
Sex	F	ref		
	M	0.98*	0.97	0.99
Disease stage	Asymptomatic	ref		
	Presymptomatic	1	0.94	1.05
	Symptomatic	2.29*	2.25	2.32
	Late Symptomatic	0.64*	0.61	0.69
	Unknown	1.08*	1.06	1.10

Coefficients on immunity status and dominant VOC were included in the Supplementary Table S2 (VOC, Variant of Concern, Age group in years; F, Female; M, Male, * = 1 outside of the 95% CI), Belgium, 29 March 2021–22 February 2022.

**Figure 2:**
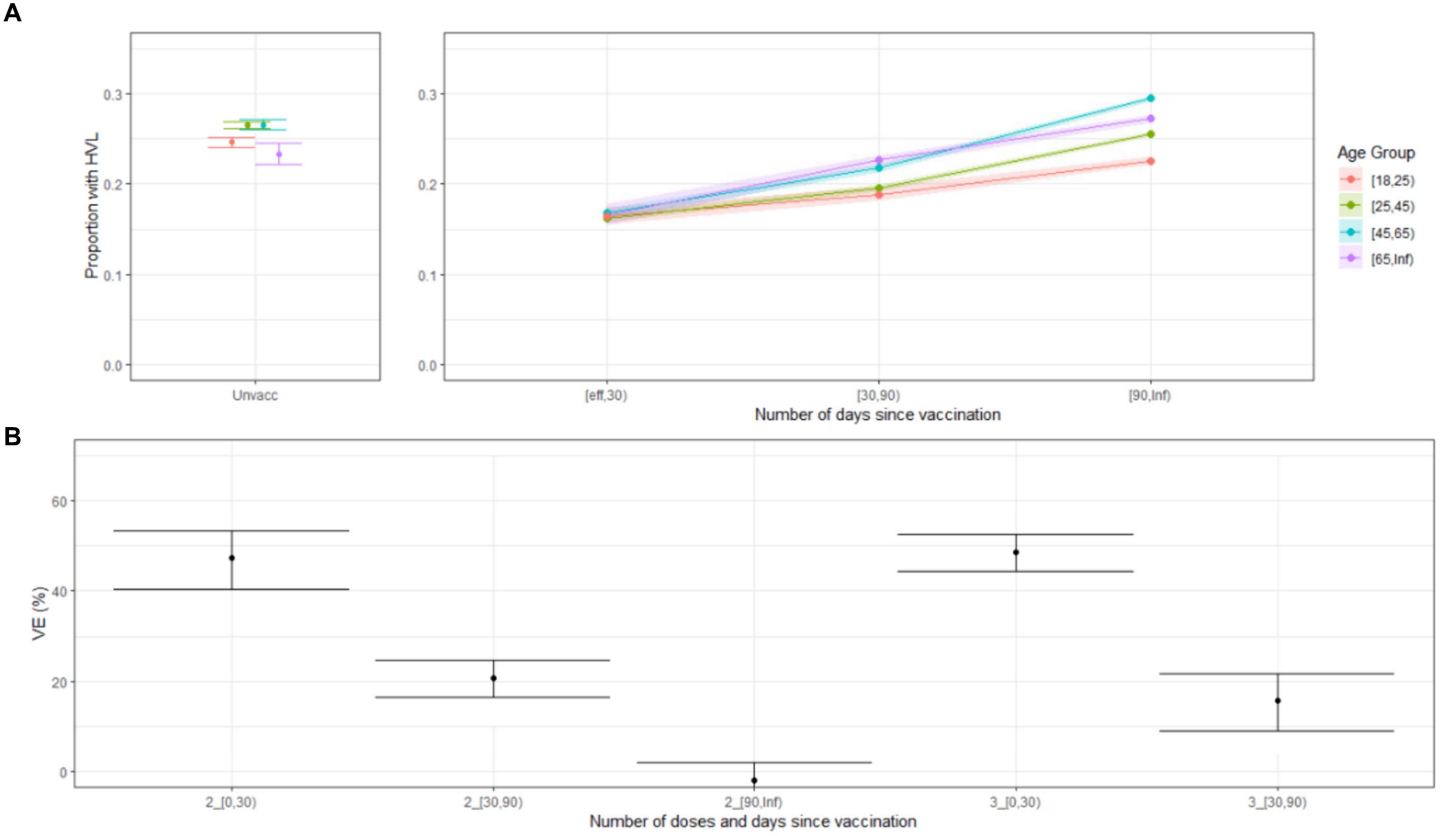
**(A)** Proportion of cases with high viral load (HVL) over all cases with SQ-PCR results, by vaccination status (unvaccinated and having received at least one dose) and time since vaccination, Delta & Omicron dominant periods, including only cases without prior infection, Belgium, 6 July 2021–22 February 2022. **(B)** Vaccine effectiveness (VE) against HVL by time since vaccination and number of doses for persons without laboratory-confirmed prior infection aged 25–44 years (reference: unvaccinated, no laboratory-confirmed prior infection, during Delta-dominant period, Omicron results were included in Supplementary Figure S3), Belgium, 6 July 2021–27 December 2021.

The proportion of vaccinated cases with HVL increased over time since vaccination. After 90 days, most age groups reached a level comparable to that of unvaccinated cases (Figure 2A; Supplementary Figure S2). Primary schedule vaccine effectiveness against HVL was estimated at 47.3% (95%CI 40.3–53.3, delta-dominant period, first 30 days after vaccination) and 29.0% (95%CI 20.6–35.7, omicron-dominant period, first 30 days after vaccination) (Figure 2B; Supplementary Figure S3).

**Figure 3:**
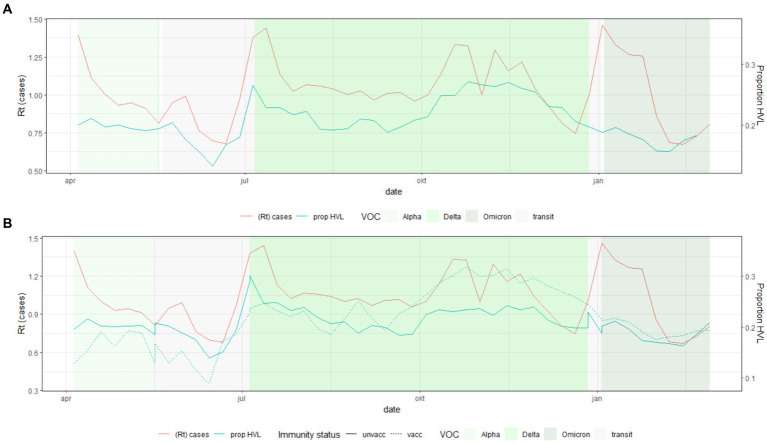
Population trends of the proportion of cases with high viral load (HVL) over all cases with SQ-PCR results [Upper **(A)**] and by vaccination status [Lower **(B)**, unvacc, unvaccinated; vacc, having received at least one dose] and Rt as estimated from overall case-incidence over time (all metrics were calculated over the 7 days prior to the presented date) (SQ, Semi-Quantitative; VOC, Variant of Concern; Rt, Real-time reproduction number), Belgium, 29 March 2021–22 February 2022.

On the population level, the proportion of cases with HVL overall and by vaccination status was associated with the general epidemiological trend. The high incidence associated with the Delta variant from October 2021 onwards was associated with an increase in the proportion of HVL, mainly in the vaccinated population. The proportion with HVL reached 30% among vaccinated persons in November 2021 (Figure 3).

### 3.3 Transmission by and of viral load

An initial, unadjusted analysis showed an association between the SQ-PCR result of the index case and the test positivity of HREC: 41.7% of HREC tested positive if the index case had HVL and 18.9% if the index case tested weakly positive. In addition, we observed a similarity between the SQ-PCR results of the index case and the SQ-PCR results of the HREC: weakly positive SQ-PCR results were observed most frequently in HREC if the index case also had a weakly positive SQ-PCR result (32.4%). Similar observations were made for the other SQ-PCR categories (Table 3).

**Table 3:** Test positivity of HREC (left, test result of HREC, pos, positive; neg, negative) and transmission of SQ-PCR results (middle, SQ-PCR results of HREC) and percentage symptomatic among positive HREC (right, self-reported during contact tracing) by SQ-PCR result of the index case (weakly positive 9.2%, moderately positive 13%, highly positive 50.5%, and very highly positive 27.3% of all SQ-PCR results of index cases) (HVL, high viral load; SQ, semi-quantitative; Belgium), 29 March 2021–22 February 2022.

SQ-PCR index	Test positivity		Transmission of SQ-PCR results				Percentage symptomatic among positive HREC
	Pos HREC	Neg HREC	Weakly positive	Moderately positive	Highly positive	Very highly positive (HVL)	
Weakly Positive	7,818 (18.9%)	33,493 (81.1%)	1,168 (32.4%)	890 (24.7%)	1,145 (31.8%)	400 (11.1%)	33.8%
Moderately Positive	16,168 (29.6%)	38,523 (70.4%)	1831 (22.4%)	2,210 (27%)	3,106 (37.9%)	1,041 (12.7%)	39.7%
Highly Positive	72,824 (40.3%)	107,706 (59.7%)	6,462 (16.1%)	9,676 (24.1%)	18,049 (44.9%)	5,967 (14.9%)	39.8%
Very Highly Positive (HVL)	46,707 (41.7%)	65,353 (58.3%)	3,497 (14.4%)	5,073 (20.9%)	9,384 (38.7%)	6,278 (25.9%)	41.1%

In the logistic regression, transmission risk seemed to be greatest when the index case was symptomatic [OR 1.43 (95%CI: 1.39–1.46)] and had HVL [OR 2.70 (95%CI: 2.62–2.79)] as compared to asymptomatic index cases without HVL. The odds increased with each subsequent SQ-PCR category (Table 4). The effect of the symptomatic presentation and SQ-PCR of the index case was large, with a 50% increase in test positivity among HREC if the index case was symptomatic with HVL compared to an asymptomatic index case without HVL (Supplementary Figure S4). The association of the SQ-PCR result of the index cases with test positivity in the HREC was greater among household contacts than outside of the household. The OR among household contacts was 2.54 (95% CI: 2.46–2.62) and 1.91 (95% CI: 1.78–2.05) among non-household contacts for symptomatic index cases with HVL (Supplementary Table S4). No significant association was observed between the disease stage of the index case and the SQ-PCR results of the HREC when adjusting for the SQ-PCR result of the index case. SQ-PCR results between HREC and index case, however, were significantly associated: the odds ratio for HVL in an HREC was 2.84 (95% CI 2.53–3.19) if the index cases also had HVL versus no HVL.

**Table 4:** Odds ratios and lower and upper bound of the 95% CI from the transmission model for testing positive (left, reference: negative test result) and for having high viral load (right, reference: positive test without high viral load) by the disease stage and SQ-PCR result of the index case; coefficients for other variables can be found in the Supplementary Table S3 (HVL, high viral load; SQ, semi-quantitative), Belgium, 29 March 2021–22 February 2022.

Variable	Level	Test positivity			Transmission of SQ-PCR results		
		Odds ratio	Lower bound	Upper bound	Odds ratio	Lower bound	Upper bound
Disease stage	Asymptomatic	ref			ref		
	Presymptomatic	1.35*	1.26	1.43	1.06	0.88	1.28
	Symptomatic	1.43*	1.39	1.46	1.03	0.96	1.11
	Late symptomatic	1.32*	1.19	1.47	0.89	0.65	1.24
	Unknown	1.15*	1.07	1.23	0.93	0.75	1.15
SQ-PCR	Weakly positive	ref			ref		
	Moderately positive	1.62*	1.56	1.67	1.15 *	1.01	1.30
	Highly positive	2.21*	2.14	2.27	1.41 *	1.26	1.58
	Very highly positive (HVL)	2.7*	2.62	2.79	2.84 *	2.53	3.19

## 4 Discussion

In this study, we used Belgian data from SARS-CoV-2 cases and contact tracing to investigate the use of SQ-PCR results for monitoring and controlling disease spread. We found that a high viral load likely indicated a recent infection. Vaccination was effective in reducing viral load, but the effectiveness decreased over time. SQ-PCR results were associated with infectiousness and were heritable: high viral loads in index cases were associated with high viral loads in HREC.

The within-person temporal dynamics of SARS-CoV-2 viral load have been a research topic since early 2020 (10). The following dynamic has been observed: a rapid increase in the days before symptom onset, a peak in viral load 2–3 days after symptom onset, followed by a gradual decline (21–24). This dynamic seems to remain despite the variant change. We did not observe a change in magnitude over the different variants. Immunization, however, might shift the peak to the fourth day of symptoms in a highly immune adult population (25). Whether or not this is linked to an earlier symptom onset is still unclear. These processes, the clinical manifestations, different immune responses, and viral multiplication all dynamically change over time. The interactions remain incompletely clear (26). Since a growing epidemic necessarily has a high proportion of recent infections and, therefore, of HVL, several studies suggested that population-level Ct-values can help forecast an epidemic’s trajectory (27–29). A study using data from one large Belgian laboratory reported that “a decrease in Ct-values, linked to an increase of recently infected people, is likely to favor spreading and goes hand in hand with an increase in the total number of cases” (30).

After adjusting for age, sex, and immunity status, we also found an association between HVL and recent infection in symptomatic cases. Similar to other studies on this topic, we lacked a reference point, such as the date of symptom onset, for asymptomatic infections. Consequently, we were unable to investigate the temporal evolution of viral load in asymptomatic cases (31, 32). Despite this limitation, when comparing symptomatic to asymptomatic cases, we observed a higher viral load in symptomatic cases. We observed lower odds of HVL for younger individuals. Multiple studies have reported a positive association between age and viral load (33, 34). A full exploration of this association should, however, include disease severity and comorbidity burden (35). Longer viral shedding has been associated with hospitalization (36).

We observed less HVL in recently vaccinated cases and in persons with a laboratory-confirmed prior infection. VE against HVL rapidly waned over time. We observed no significant VE in persons vaccinated over 90 days ago. The short-lived effect might explain why others have not found significant effects of vaccination on viral load: Levine-Tiefenbrun et al. (37) and Woodbridge et al. (38) analyzed Ct-values and reported no effectiveness over three months and 70 days, respectively, after booster or primary vaccination. Singanayagam et al. (34) reported similar peak viral loads by vaccination status, but the the median number of days since vaccination was 102.

Like other studies on contact tracing and transmission (27, 39–43), we found higher test positivity after high-risk contact with an index case with HVL, especially when the index case was symptomatic and a household contact. For earlier coronaviruses (SARS-CoV-1), exponential dose–response models have been suggested to quantify the relation between viral load and transmission risk (44). Goyat et al. suggested a comparable dose–response model with a limited risk of transmission for viral loads <10^5^ RNA copies/mL (45). Bhavnani et al. reported that only 23% of transmission events were associated with <10^5^ RNA copies/mL (43). In our study, this would correspond to weakly and moderately positive SQ-PCR results. These categories were associated with lower test positivity in HREC: <30% vs. >40% for >10^5^ RNA copies/mL. We observed the heritability of SQ-PCR results: high viral load in index cases was associated with high viral load in HREC for both symptomatic and asymptomatic index cases. To the best of our knowledge, this has only been reported by one other study on SARS-CoV-2. A prospective US study (46) linked household transmission to a high median viral load of 10^8.8^ RNA copies/mL and reported that HVL seeded other HVL infections. While we found no other studies reporting this specific observation, a shorter time to symptom onset after exposure to HVL has been reported (41), and typically faster viral load growth is correlated with a higher peak viral load (34). A Norwegian study also reported that those infected by asymptomatic cases were almost three times more likely to be asymptomatic compared to those infected by symptomatic cases (47). Delays associated with contact tracing should be further explored to investigate this finding fully, as, for example, contact tracing delays might coincide with the serial interval.

Our study contributes to an area of research where conflicting findings have been reported, particularly concerning the association between viral load and factors such as age, vaccination, or clinical presentation (10, 32, 48–51). Investigating a time-varying variable with individual heterogeneity and a large number of potential confounders required a large sample. We could include 909,157 records because of harmonization efforts by the NRC. However, our study has several limitations. While laboratories were mandated to report cases, reporting SQ-PCR results was not required. We explored the impact of missing SQ-PCR results in the Supplementary materials and found no evidence of a significant effect. However, it remains a missing data issue with the potential for biases. The data on symptoms (date of symptom onset, asymptomatic) is self-reported and mostly based on a single interview. This limitation extends to contact tracing data, which was mostly self-reported. While contact tracing linked subsequent cases by high-risk exposure, the index case was not necessarily the infector of its contacts. We used the data as reported. An in-depth description of Belgian contact tracing performance can be found elsewhere (52). Prior infections could only be included if they were ‘known,’ laboratory-confirmed prior infections. In addition, we did not include the type of vaccine (viral vector or mRNA), brand, or details on the schedule (heterological/homological). Our analysis did not try to further improve the harmonization; other factors that could influence the SQ-PCR result (sample preparation, extraction method, storage, transport) could not be controlled for. It remains important to stress that while we considered SQ-PCR results as a proxy for HVL, they are not a direct measure of viral load.

We explored the use of ordinal regression to allow all SQ-PCR categories into the transmission analyses, but the proportional odds assumption was violated. Therefore, we dichotomized the SQ-PCR results into HVL and non-HVL for some analyses. While an interest exists in the other SQ-PCR categories, this approach allowed for the use of straightforward statistical methods. We set our threshold for HVL at 10^7^. While this value can be considered at the higher end, it provided us with adequate discriminatory power. This study only allows for an adjusted, within-variant interpretation of SQ-PCR, and the results might not translate to new variants. We did not specifically discuss (rapid) antigen tests. These are often included in this type of research since their sensitivity can be linked to SQ-PCR results.

Our research has significant public health implications. A more targeted contact tracing system could prioritize cases with HVL. Given the temporal relation of HVL and symptom onset, case detection and isolation should be swift to avoid transmission. Vaccination campaigns aiming to reduce viral loads should consider the temporary nature of the effect.

## Data Availability

The data analyzed in this study is subject to the following licenses/restrictions: data on contact tracing and laboratory testing as used in this paper are not publicly available. Anonymized and aggregated datasets from the laboratory result datasets on COVID-19 are available on the Health Data Agency website (https://catalog.hda.belgium.be/). Some datasets are instantly available, for others, access has to be requested. The procedure is explained on the website. Requests to access these datasets should be directed to https://catalog.hda.belgium.be/.
