# Supplementary material for: Personal characteristics and transmission dynamics associated with SARS-CoV-2 semi-quantitative PCR test results: an observational study from Belgium, 2021–2022
Source: Front Public Health. 2024 Sep 10;12:1429021. doi: 10.3389/fpubh.2024.1429021 (PMC11420023; doi:10.3389/fpubh.2024.1429021)
Supplement: Supplementary file 1 [file Data_Sheet_1.docx]

Supplementary Materials

In this supplementary materials, we present supplementary tables, figures and analyses to the paper titled: ‘Personal characteristics and Transmission dynamics associated with SARS-CoV-2 semi-quantitative PCR test results, a proxy for viral load, Belgium, 2021-2022.’. We subsequently included the supplementary (1) tables, (2) figures, (3) analyses on the selective reporting of SQ-PCR and (4) multiple measurements of SQ-PCR over the same infection.

## Supplementary tables

Supplementary Table S1: Odds Ratios and lower and upper bounds of the 95% Confidence interval from the mixed model (within infection analysis) on data from persons with multiple SQ-PCR measurements over the same infection, Belgium, 29 March 2021 – 22 February 2022.

Tests included: 69020 Persons included: 31615

| variable | Odds Ratio | Lower bound | Upper bound |
| --- | --- | --- | --- |
| (Intercept) | 0.05 | 0.05 | 0.05 |
| Presymptomatic | 1.44 | 1.14 | 1.81 |
| Symptomatic | 3.81 | 3.56 | 4.08 |
| Late symptomatic | 0.28 | 0.25 | 0.30 |
| Unknown | 1.14 | 1.07 | 1.23 |

Supplementary Table S2: Odds ratios and lower and upper bound of the 95% CI from the analysis on personal and temporal characteristics associated with high viral load (reference: positive test without high viral load). Coefficients on dominant VOC, age, sex and disease stage were included in the main manuscript Table 2, Belgium, 29 March 2021 – 22 February 2022

| **N doses** | **Days since vaccination** | **Pr. inf** | **VOC** | **Odds Ratio** | **Lower bound** | **Upper bound** |
| --- | --- | --- | --- | --- | --- | --- |
| 0 | NA | None |  | ref |  |  |
|  |  | 60-120 | Alpha | 0.15* | 0.06 | 0.41 |
|  |  |  | Delta | 0.4* | 0.27 | 0.58 |
|  |  |  | Omicron | 1.1* | 1.01 | 1.20 |
|  |  | >120 | Alpha | 0.32* | 0.21 | 0.48 |
|  |  |  | Delta | 0.58* | 0.52 | 0.64 |
|  |  |  | Omicron | 0.87* | 0.83 | 0.91 |
| 1 | [eff,30) | None | Alpha | 0.8* | 0.70 | 0.92 |
|  |  |  | Delta | 0.86* | 0.79 | 0.92 |
|  |  |  | Omicron | 0.81* | 0.75 | 0.87 |
|  |  | 60-120 | Delta | 0.32 | 0.08 | 1.34 |
|  |  |  | Omicron | 0.43* | 0.19 | 0.99 |
|  |  | >120 | Delta | 0.33* | 0.16 | 0.69 |
|  |  |  | Omicron | 0.64 | 0.40 | 1.01 |
|  | [30,90) | None | Alpha | 0.9 | 0.76 | 1.07 |
|  |  |  | Delta | 0.91* | 0.85 | 0.98 |
|  |  |  | Omicron | 0.96 | 0.84 | 1.10 |
|  |  | 60-120 | Delta | 0.1* | 0.01 | 0.76 |
|  |  |  | Omicron | 1.38 | 0.56 | 3.39 |
|  |  | >120 | Delta | 0.34* | 0.17 | 0.65 |
|  |  |  | Omicron | 0.58* | 0.38 | 0.89 |
| 2 | [eff,30) | None | Alpha | 0.52* | 0.30 | 0.91 |
|  |  |  | Delta | 0.47* | 0.41 | 0.54 |
|  |  |  | Omicron | 0.68* | 0.61 | 0.76 |
|  |  | 60-120 | Delta | 0.16 | 0.02 | 1.21 |
|  |  |  | Omicron | 0.66 | 0.23 | 1.87 |
|  |  | >120 | Delta | 0.16* | 0.05 | 0.50 |
|  |  |  | Omicron | 0.57* | 0.36 | 0.90 |
|  | [30,90) | None | Alpha | 0.45* | 0.32 | 0.62 |
|  |  |  | Delta | 0.75* | 0.72 | 0.79 |
|  |  |  | Omicron | 0.85* | 0.82 | 0.89 |
|  |  | 60-120 | Delta | 0.15* | 0.05 | 0.47 |
|  |  |  | Omicron | 0.93 | 0.61 | 1.40 |
|  |  | >120 | Alpha | 0.08* | 0.01 | 0.57 |
|  |  |  | Delta | 0.31* | 0.22 | 0.43 |
|  |  |  | Omicron | 0.66* | 0.56 | 0.78 |
|  | [90,Inf) | None | Delta | 1.02* | 1.00 | 1.05 |
|  |  |  | Omicron | 0.97* | 0.95 | 0.99 |
|  |  | 60-120 | Delta | 0.33* | 0.18 | 0.58 |
|  |  |  | Omicron | 1.05 | 0.91 | 1.21 |
|  |  | >120 | Delta | 0.56* | 0.51 | 0.62 |
|  |  |  | Omicron | 0.78* | 0.74 | 0.82 |
| 3 | [eff,30) | None | Delta | 0.46* | 0.42 | 0.50 |
|  |  |  | Omicron | 0.61* | 0.59 | 0.63 |
|  |  | 60-120 | Omicron | 0.6* | 0.44 | 0.81 |
|  |  | >120 | Delta | 0.35* | 0.19 | 0.63 |
|  |  |  | Omicron | 0.6* | 0.54 | 0.68 |
|  | [30,90) | None | Delta | 0.81* | 0.75 | 0.88 |
|  |  |  | Omicron | 0.81* | 0.79 | 0.83 |
|  |  | 60-120 | Delta | 0.37 | 0.05 | 3.03 |
|  |  |  | Omicron | 0.61* | 0.50 | 0.75 |
|  |  | >120 | Delta | 0.16* | 0.06 | 0.43 |
|  |  |  | Omicron | 0.61* | 0.56 | 0.66 |
|  | [90,Inf) | None | Delta | 0.93 | 0.60 | 1.43 |
|  |  |  | Omicron | 1.01 | 0.97 | 1.05 |
|  |  | 60-120 | Omicron | 0.53* | 0.32 | 0.88 |
|  |  | >120 | Delta | 0.68 | 0.07 | 6.67 |
|  |  |  | Omicron | 0.64* | 0.55 | 0.73 |

Supplementary Table ST3: Odds Ratios and upper and lower bound of the 95% confidence interval from the transmission model for HREC testing positive (P(pos HREC), reference: HREC testing negative) associated with covariates N.HREC (total number of HREC reported by the index case that reported the HREC for which we fit the test result), N.exposures (Number of different index cases reporting the HREC for which we fit the test result), VOC (dominant VOC), household (0=non-household partners, 1=household partners, UNK=unknown), CIAH (case previously identified as HREC, the index case was known to contact tracing as he/she had been previously (last 20 days) been identified as HREC), age group, sex and immunity status for the index case and HREC. Coefficients for disease stage and SQ-PCR result can be found in the main manuscript Table 4, SQ=Semi-quantitative, Belgium, 29 March 2021 – 22 February 2022

| **Variable** | **Level** | **Odds Ratio** | **Lower bound** | **Upper bound** |
| --- | --- | --- | --- | --- |
| N.HREC |  | 0.98* | 0.98 | 0.98 |
| N.exposures |  | 1.26* | 1.23 | 1.29 |
| VOC | Alpha | ref |  |  |
|  | Delta | 0.98 | 0.81 | 1.18 |
|  | Omicron | 0.39* | 0.33 | 0.47 |
|  | transit | 0.66* | 0.56 | 0.78 |
| household | 0 | ref |  |  |
|  | 1 | 2.75* | 2.69 | 2.81 |
|  | UNK | 1.63* | 1.57 | 1.70 |
| CIAH | gen0 | ref |  |  |
|  |  | 0.93* | 0.91 | 0.96 |

|  |  | **Index case** | | | **High-Risk Exposure Contact (HREC)** | | |
| --- | --- | --- | --- | --- | --- | --- | --- |
| **variable** | **level** | **Odds ratio** | **Lower bound** | **Upper bound** | **Odds Ratio** | **Lower bound** | **Upper bound** |
| Age group | [00,06) | 0.84* | 0.80 | 0.88 | 0.55* | 0.53 | 0.57 |
|  | [06,18) | 0.73* | 0.71 | 0.74 | 0.82* | 0.80 | 0.84 |
|  | [18,25) | 0.73* | 0.71 | 0.75 | 0.83* | 0.81 | 0.86 |
|  | [25,45) | ref |  |  | ref |  |  |
|  | [45,65) | 1.11* | 1.09 | 1.14 | 0.89* | 0.88 | 0.91 |
|  | [65,Inf) | 1.49* | 1.43 | 1.56 | 0.96 | 0.93 | 1.00 |
| Sex | F | ref |  |  | ref |  |  |
|  | M | 1.04* | 1.03 | 1.06 | 0.94* | 0.93 | 0.96 |

|  |  |  |  | **Index case** | | | **High-Risk Exposure Contact (HREC)** | | |
| --- | --- | --- | --- | --- | --- | --- | --- | --- | --- |
| **VOC** | **N doses** | **Days since vaccination** | **Pr. inf** | **Odds Ratio** | **Lower bound** | **Upper bound** | **Odds Ratio** | **Lower bound** | **Upper bound** |
|  | 0 | NA | 0 | ref |  |  | ref |  |  |
| Alpha |  |  | 1 | 0.6* | 0.42 | 0.84 | 0.17* | 0.13 | 0.22 |
|  | 1 | [eff,30) | 0 | 0.9 | 0.76 | 1.05 | 0.78* | 0.68 | 0.90 |
|  |  | [30,90) |  | 0.77* | 0.63 | 0.93 | 0.49* | 0.42 | 0.57 |
|  | 2 | [eff,30) |  | 0.37* | 0.17 | 0.81 | 0.2* | 0.14 | 0.29 |
|  |  | [30,90) |  | 0.43* | 0.30 | 0.63 | 0.19* | 0.15 | 0.24 |
|  |  | [90,Inf) |  | 0.17* | 0.04 | 0.72 | 0.31* | 0.14 | 0.70 |
| Delta | 0 | NA | 1 | 0.62* | 0.55 | 0.70 | 0.26* | 0.24 | 0.29 |
|  | 1 | [eff,30) | 0 | 0.8* | 0.73 | 0.87 | 0.5* | 0.46 | 0.55 |
|  |  | [30,90) |  | 1.03 | 0.96 | 1.12 | 0.39* | 0.36 | 0.42 |
|  |  | [90,Inf) |  | 0.79* | 0.74 | 0.84 | 0.5* | 0.47 | 0.54 |
|  |  | [eff,30) | 1 | 0.95 | 0.45 | 2.03 | 0.08* | 0.04 | 0.14 |
|  |  | [30,90) |  | 0.27* | 0.10 | 0.72 | 0.1* | 0.06 | 0.17 |
|  |  | [90,Inf) |  | 1.09 | 0.68 | 1.74 | 0.08* | 0.05 | 0.12 |
|  | 2 | [eff,30) | 0 | 0.84* | 0.74 | 0.95 | 0.16* | 0.15 | 0.18 |
|  |  | [30,90) |  | 0.73* | 0.70 | 0.77 | 0.23* | 0.22 | 0.24 |
|  |  | [90,Inf) |  | 0.8* | 0.78 | 0.83 | 0.39* | 0.38 | 0.40 |
|  |  | [eff,30) | 1 | 0.6 | 0.28 | 1.30 | 0.06* | 0.03 | 0.11 |
|  |  | [30,90) |  | 0.61* | 0.44 | 0.85 | 0.09* | 0.07 | 0.11 |
|  |  | [90,Inf) |  | 0.71* | 0.63 | 0.80 | 0.11* | 0.10 | 0.12 |
|  | 3 | [eff,30) | 0 | 0.65* | 0.58 | 0.72 | 0.18* | 0.16 | 0.19 |
|  |  | [30,90) |  | 0.73* | 0.64 | 0.82 | 0.2* | 0.18 | 0.22 |
|  |  | [90,Inf) |  | 0.66 | 0.38 | 1.15 | 0.26* | 0.16 | 0.44 |
|  |  | [eff,30) | 1 | 0.94 | 0.53 | 1.67 | 0.05* | 0.03 | 0.09 |
|  |  | [30,90) |  | 0.35 | 0.10 | 1.21 | 0.08* | 0.04 | 0.16 |
| Omicron | 0 | NA |  | 0.73* | 0.67 | 0.80 | 0.65* | 0.60 | 0.70 |
|  | 1 | [eff,30) | 0 | 0.95 | 0.86 | 1.05 | 0.93 | 0.83 | 1.06 |
|  |  | [30,90) |  | 0.79 | 0.55 | 1.13 | 0.6* | 0.45 | 0.80 |
|  |  | [90,Inf) |  | 0.92 | 0.78 | 1.09 | 0.91 | 0.78 | 1.06 |
|  |  | [eff,30) | 1 | 0.54* | 0.29 | 0.98 | 0.14* | 0.09 | 0.21 |
|  |  | [30,90) |  | 1.13 | 0.54 | 2.37 | 0.17* | 0.09 | 0.30 |
|  |  | [90,Inf) |  | 0.7 | 0.48 | 1.01 | 0.42* | 0.31 | 0.58 |
|  | 2 | [eff,30) | 0 | 0.67* | 0.55 | 0.81 | 0.54* | 0.47 | 0.62 |
|  |  | [30,90) |  | 0.89* | 0.80 | 0.98 | 0.75* | 0.69 | 0.82 |
|  |  | [90,Inf) |  | 0.85* | 0.82 | 0.88 | 0.93* | 0.89 | 0.97 |
|  |  | [eff,30) | 1 | 0.44 | 0.17 | 1.16 | 0.18* | 0.11 | 0.27 |
|  |  | [30,90) |  | 0.47* | 0.34 | 0.66 | 0.35* | 0.28 | 0.45 |
|  |  | [90,Inf) |  | 0.64* | 0.58 | 0.70 | 0.44* | 0.41 | 0.48 |
|  | 3 | [eff,30) | 0 | 0.69* | 0.64 | 0.73 | 0.47* | 0.45 | 0.50 |
|  |  | [30,90) |  | 0.77* | 0.72 | 0.81 | 0.56* | 0.54 | 0.59 |
|  |  | [90,Inf) |  | 0.92 | 0.81 | 1.04 | 0.74* | 0.66 | 0.83 |
|  |  | [eff,30) | 1 | 0.51* | 0.40 | 0.66 | 0.24* | 0.20 | 0.27 |
|  |  | [30,90) |  | 0.55* | 0.46 | 0.65 | 0.27* | 0.24 | 0.31 |
|  |  | [90,Inf) |  | 1.05 | 0.53 | 2.05 | 0.33* | 0.22 | 0.50 |

Supplementary Table S4: Odds Ratios and lower and upper bounds of the 95% Confidence Intervals for the combined effect of symptoms and HVL compared between: analysis on all records (Left), analysis restricted to non-household contacts (Middle), analysis restricted to household contacts (Right), Belgium, 2021.

Included household contacts: 257814, non-household contacts: 102156

|  |  | All High-Risk Exposure Contacts (HREC) | | | Non-household HREC | | | Household HREC | | |
| --- | --- | --- | --- | --- | --- | --- | --- | --- | --- | --- |
| Symptoms | HVL | Odds Ratio | Lower bound | Upper bound | Odds Ratio | Lower bound | Upper bound | Odds Ratio | Lower bound | Upper bound |
| Asymptomatic | No | Ref |  |  |  |  |  |  |  |  |
|  | HVL | 1.75 | 1.66 | 1.85 | 1.49 | 1.28 | 1.74 | 1.80 | 1.69 | 1.91 |
| Symptomatic | No | 1.71 | 1.67 | 1.76 | 1.47 | 1.37 | 1.57 | 1.78 | 1.73 | 1.83 |
|  | HVL | 2.38 | 2.31 | 2.44 | 1.91 | 1.78 | 2.05 | 2.54 | 2.46 | 2.62 |
| Unknown | No | 1.43 | 1.32 | 1.55 | 1.53 | 1.05 | 2.23 | 1.53 | 1.35 | 1.73 |
|  | HVL | 1.72 | 1.54 | 1.91 | 1.51 | 0.83 | 2.74 | 2.28 | 1.87 | 2.78 |

## Supplementary figures


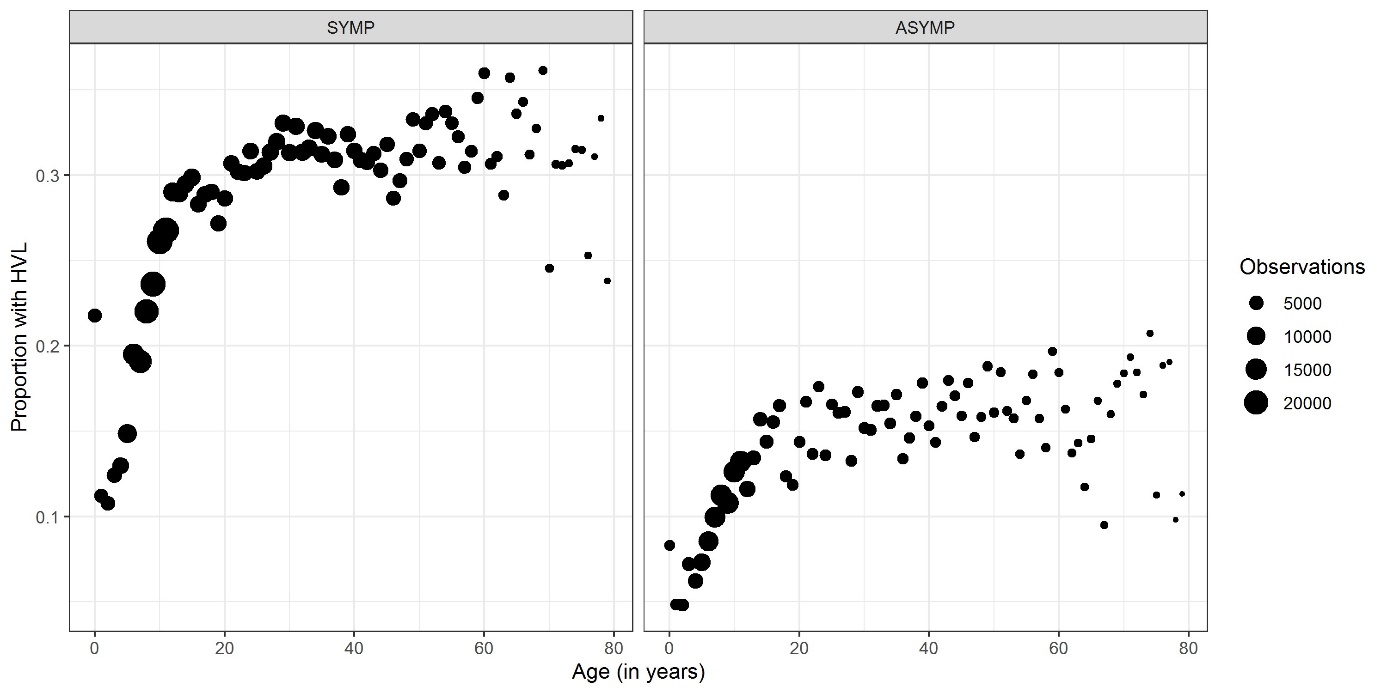


Supplementary Figure SF1: Proportion of cases with High Viral Load (HVL) over all cases with SQ-PCR results by age (in years) and symptomatic state (reporting symptoms (Left) vs not-reporting symptoms (Right)) in immune naïve individuals (no vaccination, no laboratory-confirmed prior infection). The size of the dot represents the number of observations, Belgium, 29 March 2021 – 22 February 2022.


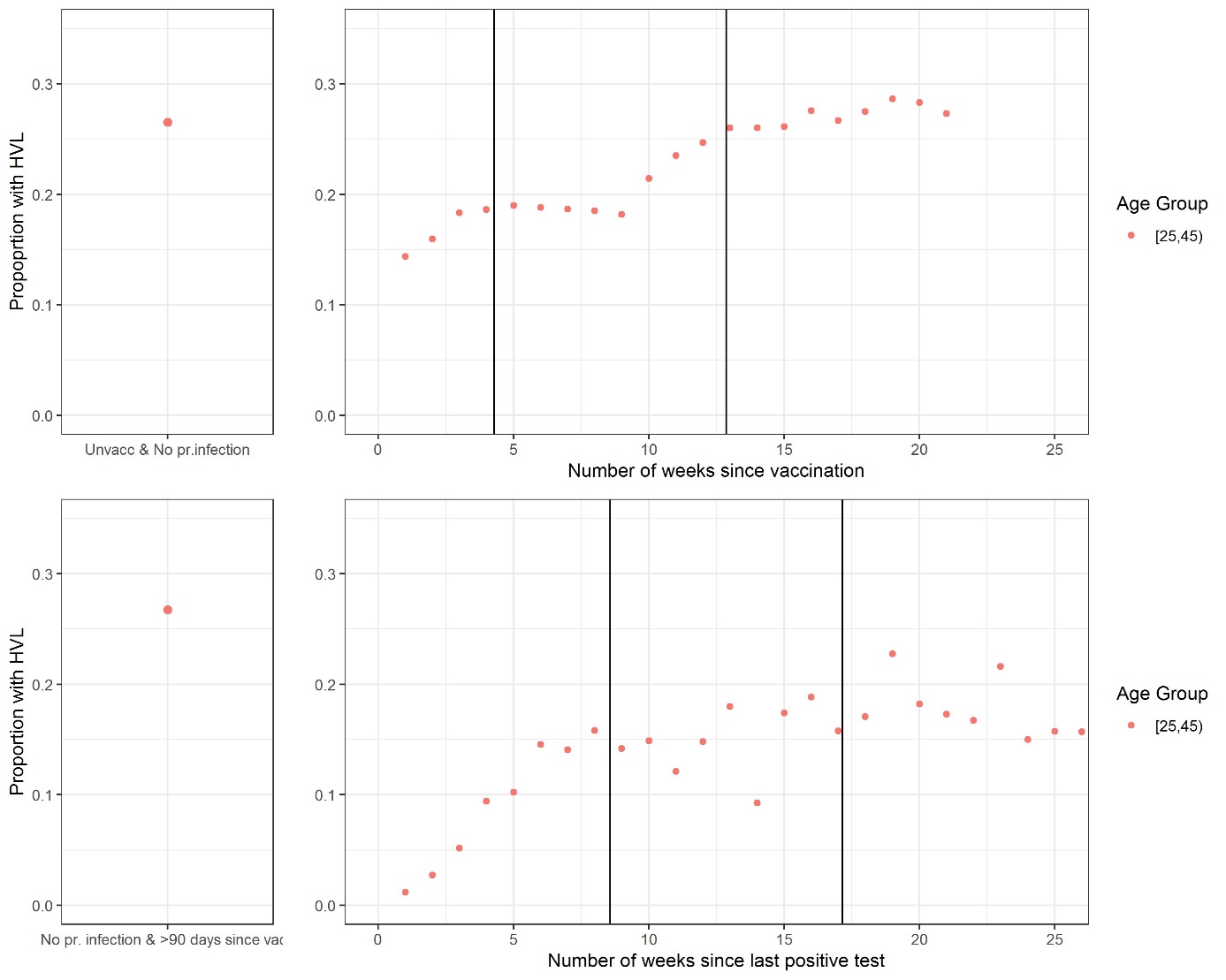


Supplementary Figure SF2: (Left, Upper) Proportion of cases with High Viral Load (HVL) over all cases with SQ-PCR results in unvaccinated persons without laboratory-confirmed prior infection, (Left, Lower) Proportion HVL in persons not recently (<90 days) vaccinated, without laboratory-confirmed prior infection (Right, Upper) Proportion HVL in vaccinated persons over the number of weeks since vaccination, (Right, Lower) Proportion HVL in persons with a laboratory-confirmed prior infection without recent (<90 days) vaccinated by number of weeks since most recent laboratory-confirmed prior infection, 25-44 years, Delta-period, Belgium, 6 July 2021 - 27 December 2021.


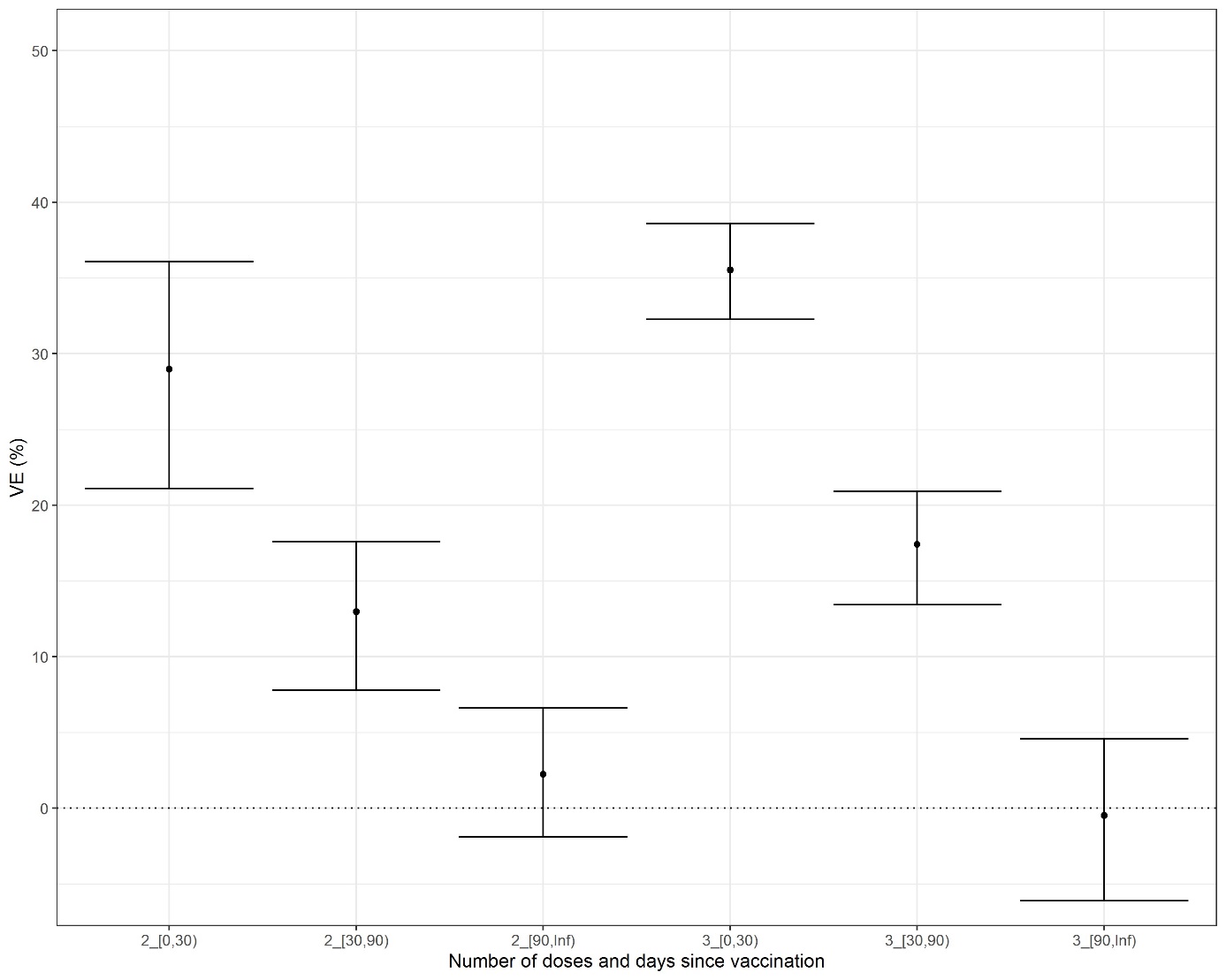


Supplementary Figure SF3: Vaccine effectiveness (VE) against HVL by time since vaccination and number of doses for persons without laboratory-confirmed prior infection aged 25-44 years (reference: unvaccinated, no laboratory-confirmed prior infection, during Omicron-dominant period, Delta results were included in the main manuscript Figure 2), Belgium, 4 January 2022 - 22 February 2022.


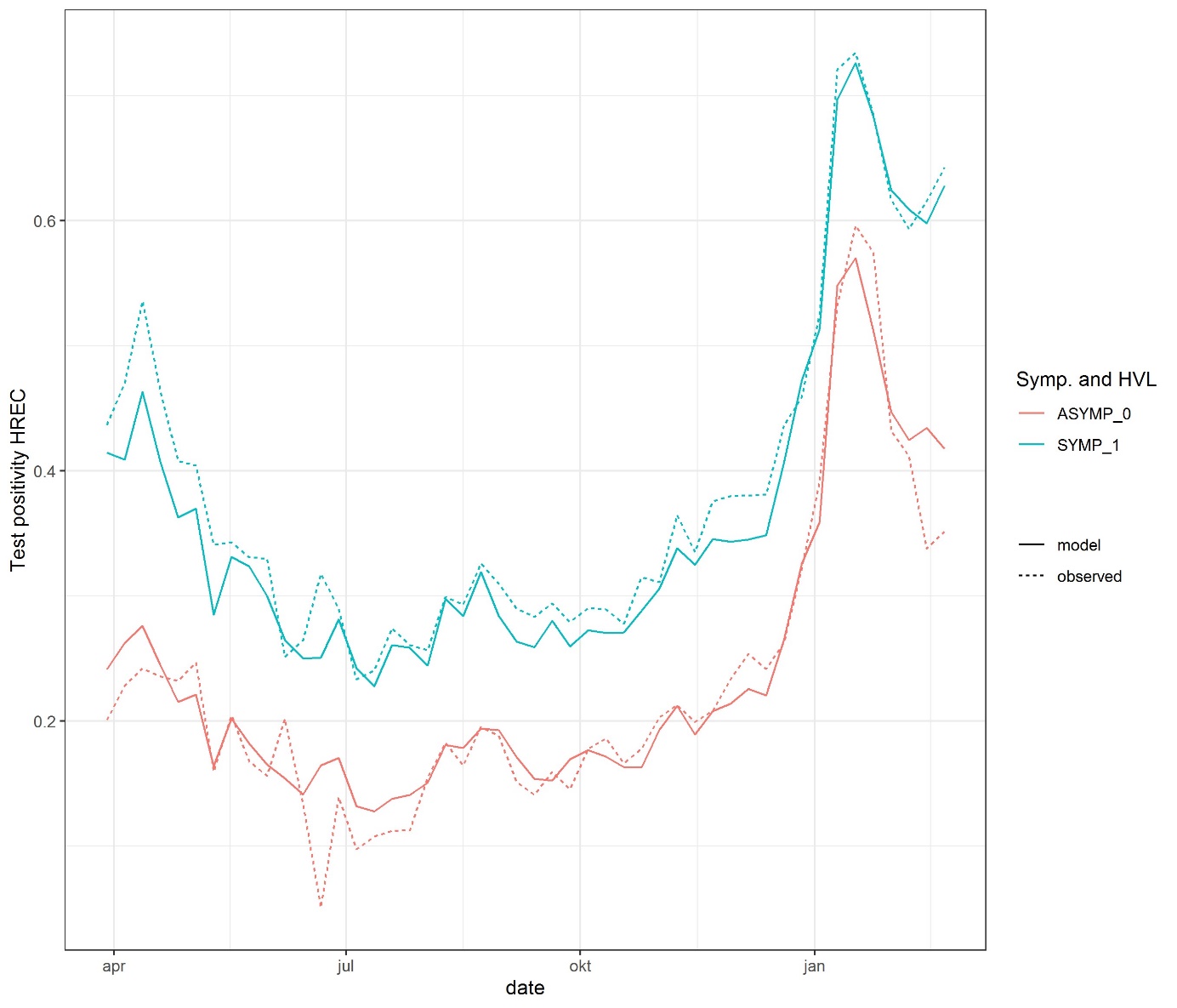


Supplementary Figure SF4: Test positivity of HREC over time observed (dotted) and predicted (full) from the transmission model by SQ-PCR result (HVL=1, no HVL=0) and symptoms of the index case (ASYMP= asymptomatic, SYMP=symptomatic), Belgium, 6 July 2021 - 27 December 2021.


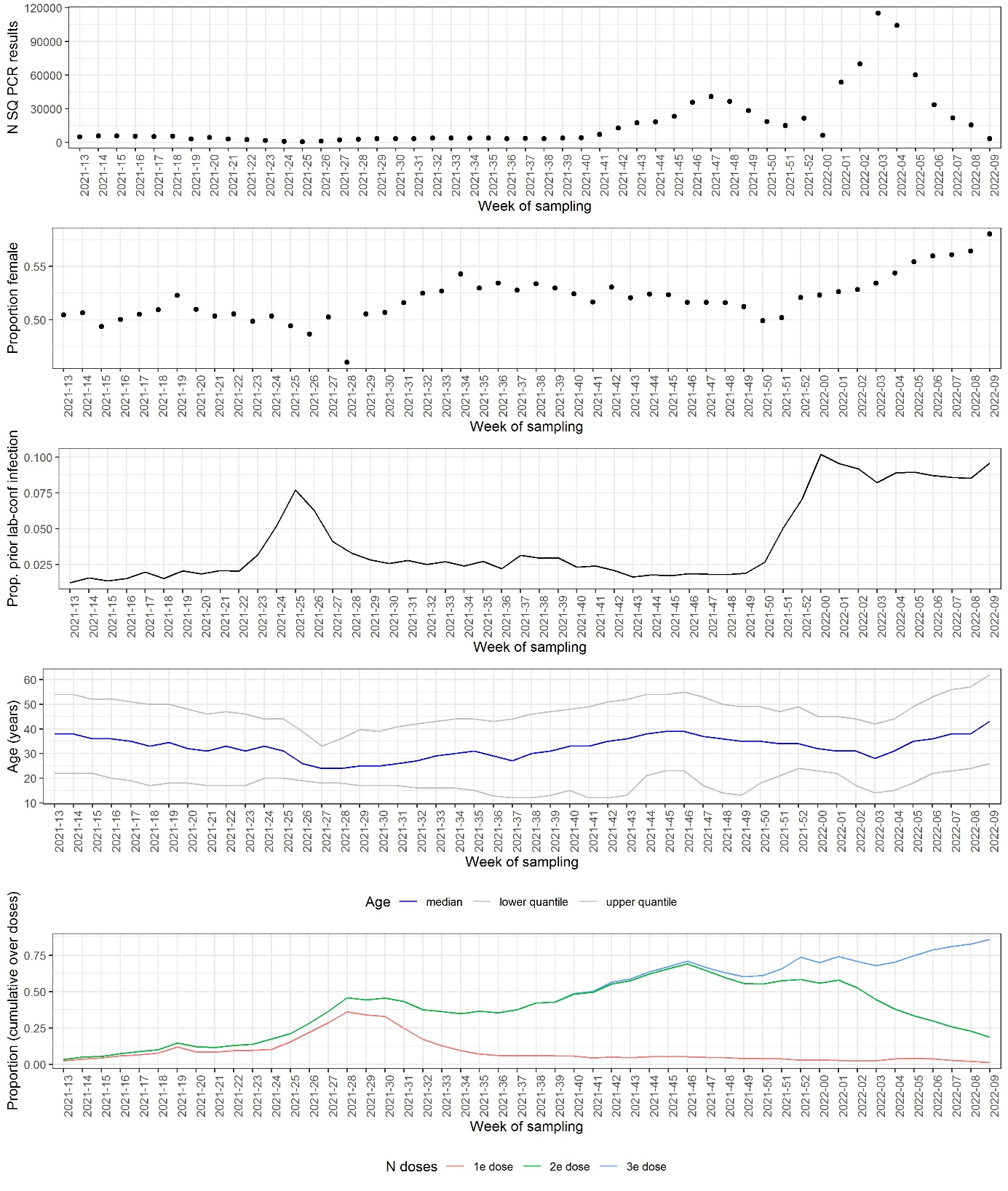


Supplementary Figure SF5: Overview of the study population (cases with SQ-PCR results). (1) Number of SQ-PCR results per day, (2) proportion of females among persons with SQ-PCR results, (3) proportion of persons with a laboratory-confirmed prior infection among persons with SQ-PCR results, (4) lower quartile, median and higher quartile for the age of persons at the time of SQ-PCR result, (5) Proportion by cumulative number of vaccine doses received by the time of SQ-PCR result, Belgium, 29 March 2021 – 22 February 2022.

## Exploration of a possible bias from selective reporting of SQ-PCR results

Not all laboratories reported SQ-PCR-results, 69% of laboratories reported no SQ-PCR results (responsible for 63% of results). For the 31% of laboratories (responsible for 37% of results) that reported SQ-PCR results, 15% started early, from April 2021 onwards, with the reporting of SQ-PCR results (responsible for 24% of results). 8% of laboratories (responsible for 10% of results) started reporting later (typically from October 2021 or from January 2022). As a general rule, the early and late reporters reported SQ-PCR results for over 90% of their tests from their start onwards. 8% of laboratories (responsible for 3% of results) reported SQ-PCR results for some of their test results without achieving 90% consistently.

For those laboratories, that reported SQ-PCR results for only a proportion of their tests (inconsistent reporters), we could not identify a clear association with the reason for prescription. Because of the low number of tests associated with these nine laboratories more in depth exploration, such as reporting SQ-PCR by age given prescription reason, was not performed as it was unlikely to affect the overall results and preliminary exploration did not indicate selective reporting shared by the laboratories.

The different start date of early and late laboratories did introduce temporal variation. The proportion of SQ-PCR results increased over time. Interestingly, differences in the test profile and regions of early and late laboratories introduced additional trends. Early laboratories were associated with Flanders and Brussels: 35% of the results from Flanders were from early reporters, 19% from the Brussels, only 3% from Wallonia. Given that there were regional differences in testing and tracing this introduced temporal trends.

Among cases, 38% of previous HREC had a SQ-PCR result, for non-HREC only 31% had a SQ-PCR result. Part of the discrepancy was found in differences between laboratories reporting SQ-PCR results and those not reporting SQ-PCR results. Among early and late reporters the proportion of HREC were 17% and 16% while only 12% among those not reporting. These differences were present also as regional differences: in Flanders 16% of new cases were previously HREC, in Brussels 15%, only 9.5% in Wallonia. Given the laboratory, SQ-PCR was as likely reported for HREC and primary cases. Within region differences were small. E.g. for Flanders SQ-PCR results for 34% of primary cases and for 36% of HREC

A similar observation was made with respect to symptoms: 25% of tests from the early reporters were from asymptomatic persons, while late reporters reported 17% asymptomatic persons. The proportion of symptomatic cases with a SQ-PCR result increased over time as late reporters started reporting.

In conclusion, only a proportion of cases had an SQ-PCR result, but we expect no bias associated with selective reporting. Given the laboratory we did not identify differences between cases with and without SQ-PCR result. Overall trends however should be adjusted by early/late/zero reporting laboratories as there were differences in testing and tracing between laboratories typically linked to the regional testing and tracing policies.

## Multiple measurements

When considering multiple SQ-PCR tests, we preferably kept the HVL SQ-PCR test (if available). Only few records were affected by this procedure and, consequently, results were relatively unaffected (in comparison to removing observations with multiple SQ-PCR tests).

For the person-level analysis, we had 15,718 individual disease stages for which multiple, different SQ-PCR were available. For 4,550 disease stage, one of the SQ-PCR tests indicated HVL. Therefore only 4,550 observations were affected by preferably keeping the HVL result.

For the transmission analysis, we had 8,049 HREC with multiple and different SQ-PCR results. Of these 1,021 included one HVL observations and were affected by the choice to retain the HVL observation.
